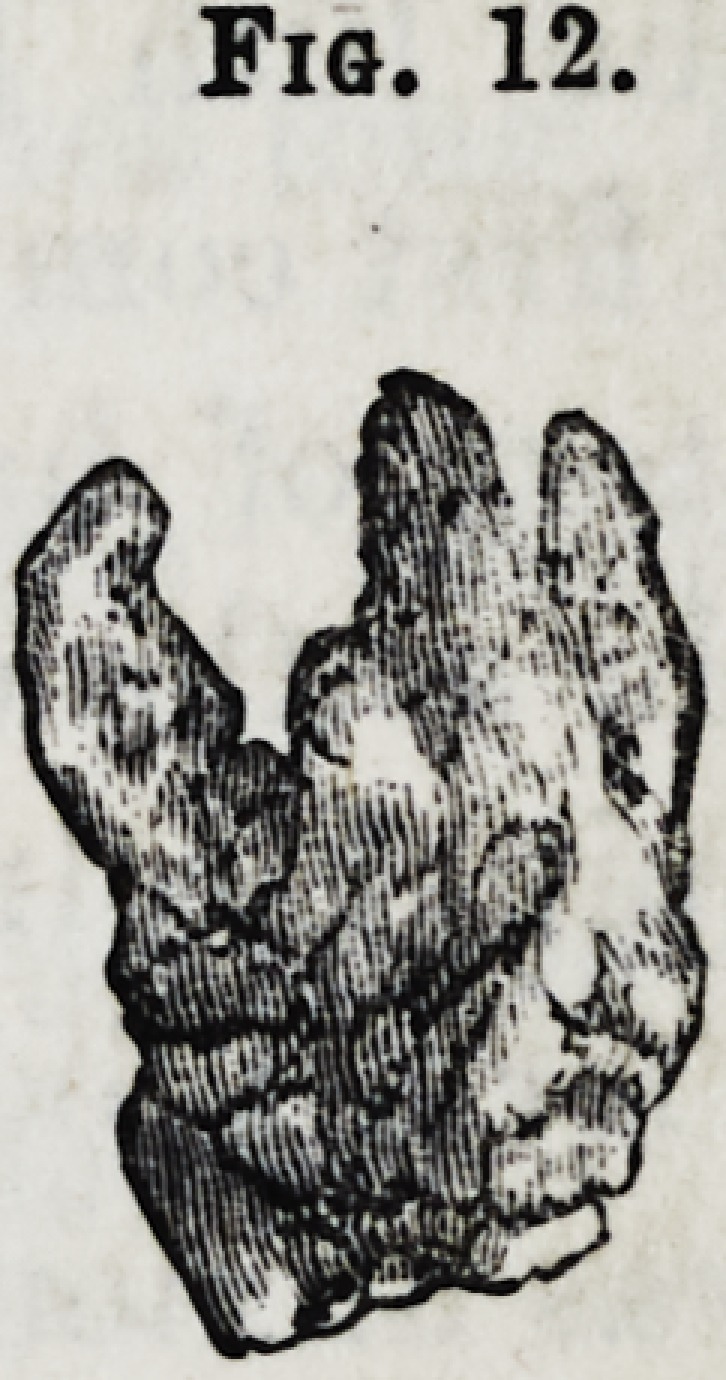# The Adapted Forceps

**Published:** 1858-04

**Authors:** J. Robinson


### SELECTED ARTICLES.

### ARTICLE X.

*The Adapted Forceps.* By J. Robinson, Esq.

Molars are much more firmly articulated, and having diverging fangs, require a much greater amount of mechanical force for their extraction.

When the proper sized forceps have been selected, they should be applied as high up under the gum as possible ; the force to be used should be first external, then internal, and so on in quick succession ; the greater amount of force should however be given to the outward movement rather than to the inward, or otherwise one or both of the buccal fangs of these teeth are likely to be fractured, particularly if either are curved at the apex of the fang; during these alternate and lateral motions, the operator must not forget the necessary perpendicular action: the alternate motions being designed to separate the tooth from the adhesions to the alveolar process, whilst the downward or perpendicular action is to draw it from the socket.

The *dentes sapientiæ* are extracted by movements similar to those employed for the first and second molars, with this exception, that these teeth being situated at the angles of the jaw, and their fangs not being generally so deeply seated in the socket, the operator should use a greater amount of force externally during the operation of extraction. He should also be particular when he applies the forceps, to observe that they are forced *well up under* the gum, and be careful that in the application of his instrument, that he does not enclose the gum and alveolar process either externally or internally within the beaks of his forceps; if so, it is more than possible a large portion of alveolar process will be brought away with the tooth, which may be followed by a protracted hemorrhage, frequently difficult to arrest, as it is within the immediate vicinity of the dental canals and artery. Moreover loose portions of bone will invariably remain, producing intense local sufferings from inflammation, suppuration and exfoliation.

*Central Incisors, Lower Jaw.*—Having in the preceding observations confined my description to the extraction of teeth from the upper jaw, I will now commence with the four central incisors of the lower jaw, premising that I differ from other practitioners as regards the shape of the instrument to be used for these teeth. As most dentists, both in England and America, use small straight-beaked forceps, I prefer the narrow-beaked hawk's-bill, excepting in those cases of irregularity, when either of these teeth are developed within or without the dental circle; in such cases the narrow pointed straight forceps should be employed. The proper instrument having been selected and applied to the tooth to be extracted, the alternate, lateral, and *strong* perpendicular motions should be used for their extraction, taking particular care, however, in the selecting the forceps, that the beaks are not too wide to cause injury to the adjoining teeth.

*Canines and Bicuspids.*—Either of these teeth can be extracted in ordinary cases with the proper sized hawk's-bill forceps, the motions being precisely the same as the preceding.

In deeply seated stumps of these teeth, hollowed out by caries, or where only a small portion of the crown remains, to resist the pressure of the instrument, the use of the elevator becomes necessary, which I shall more particularly describe hereafter.

*The Molars.*—These teeth require the alternate lateral and perpendicular movements for their extraction; the external more particularly should predominate, inasmuch as the external alveoli plate being thinner, yields more readily to the force applied.

*Dentes Sapientiæ.*—These teeth generally are easily removed with the proper forceps, particularly when well developed, and a sufficiency of crown appears above the gum to admit of a firm grip with the instrument. The motions should be alternate and lateral, combined with a slight perpendicular and horizontal action. As these teeth are usually developed at the angle, or curvature of the jaw, the fangs are frequently curved, and turned towards the coronoid process.

In many cases that occur in practice, and particularly those in which one side of the tooth has been destroyed by caries, the use of the forceps becomes uncertain; in such cases I prefer using the elevator to running the risk of a fracture with the forceps, and afterwards being compelled to have recourse to the former instrument; in fact many cases will present themselves to the pupil, in which the buccal side of the crown has been destroyed by caries, where the key instrument can be used with satisfactory results, the fulcrum of the instrument being placed upon the buccal side of the tooth.

These teeth are frequently curved in their fangs, and developed in malpositions in the jaw, and being in the immediate proximity of the inferior dental artery, render hemorrhage very common after extraction; and several cases have been reported in which fatal results have followed their extraction; but of these I shall more particularly refer when speaking of the styptics employed, and the mechanical treatment for arresting dental hemorrhage.

*Some Cases that occur in Practice.*—In the upper jaw, supernumerary teeth will be developed, either between the central incisors or high up in the anterior part of the palate; they are generally cone shaped, with a single fang, and are easily extracted with a pair of straight forceps—the same mechanical movements being employed as those used for the extraction of the upper centrals and laterals. In some cases the upper canines are occasionally externally developed high up in the alveolus, disfiguring the contour of the face; in many cases mechanical ingenuity has failed in bringing them into a regular position, particularly if they have been developed late, and made their appearance immediately over the laterals, the bicuspids having arranged themselves by the sides of the former, their extraction in many such cases becomes an imperative necessity.

Fig. 1.

When the crowns of these teeth have protruded sufficiently through the gum to allow of a firmer grip, they can be extracted by a strong but finely-pointed straight forceps in the usual way, the operator taking particular care to run the points of his instrument well up under the gum so as to obtain as firm a grasp as possible.

In making the alternate lateral motions, the external should predominate with the perpendicular, to prevent injury to the gum and the teeth beneath these irregular teeth.

Occasionally the second bicuspid will make its appearance within the palatine arch, its labial surface being closely in contact with the first bicuspid and first molar, leaving no room for the insertion of our ordinary forceps.

To meet these cases I have constructed the forceps figured at page 71 in this Journal, the finer beak of the forceps being intended to pass up, and between the molar and first bicuspid, while the inner or palatine beak is of the usual breadth, and embraces the palatine neck of the tooth. These forceps are intended for the left upper jaw, but the same instrument is also applicable to the right side of the lower jaw under similar circumstances.

Fig. 2.

In the lower jaw, it will frequently be found, that one of the incisors has been developed within the dental circle, fig. 2, the adjoining teeth being closely arranged in regular order, the irregular tooth is not only disfiguring, but it causes an obstruction to the free movement of the tongue, and its removal becomes an absolute necessity. For this purpose, as I before stated, the narrow, but strongly pointed forceps should be used, fig. 3, the operator standing behind

Fig. 3.

his patient, and supporting the chin with the left hand, while using the ordinary motions for extracting. If the point of the forceps will not embrace the anterior neck of the irregular tooth, owing to its close proximity to the posterior surfaces of the other incisors, the operator must use a pair of forceps more bowed than those figured, so as to clear the cutting edge of the tooth, and grasp the tooth sideways close down to the gum, taking care, however, not to use too great a pressure when he applies the instrument, or the tooth may be fractured, rendering the subsequent operation both difficult and tedious.

The canines in the lower jaw are sometimes developed without the dental circle, and in cases where their position is low down, so as to disfigure the contour of the mouth, without the possibility of being brought into regular order by mechanical means, their removal should be at once decided upon. If there be sufficient space between the anterior surface of the incisor or first bicuspid, and the posterior surface of the irregular tooth, the operator should use the fine straight pointed forceps, or the narrow-beaked hawk's-bill; if the former, his position for operating should be behind the patient, supporting the chin with his left hand. The motion should be external and perpendicular, carefully avoiding an inward movement, and the consequent injury to the adjoining teeth. If the hawk's-bill be employed, the operator should stand in front or on one side of his patient. If the posterior surface of the irregular tooth be in contact with the other teeth, so as to prevent the application of the instrument in that manner, the straight-bowed pointed forceps, or the hawk's-bill, can be applied laterally, using the alternate lateral and perpendicular motions, steadily, but firmly. The same teeth, when developed within the dental circle, and their removal necessary, they can be extracted by a similar movement, the straight forceps being usually employed. The bicuspids, when protruding externally through the gum, and low down in the alveolar process, can be easily removed with the hawk's-bill, or the straight or curved forceps. When developed internally the instrument I have previously referred to can be employed.

The fangs of the first and second molars in the lower jaw, are sometimes, from caries or from accidental fracture in operating, are left in the jaw to within a line of the edge of the gum, frequently retaining a small portion of bone, uniting the two fangs, but not sufficient to withstand the pressure of our ordinary forceps, so as to ensure the extraction of both fangs at once. The following exhibits a section of a first, second, and third molar, the second having been fractured, fig. 4; in such cases I have frequently di-

FIG. 4.

FIG. 5.

vided the fangs with these kind of forceps, fig. 5, which it will be observed has two cutting spines, meeting within half a line or so of each other, the other parts of the beaks being grooved in the same manner as the ordinary lower forceps; the object of which is, whilst using the instrument for dividing the fangs, and cutting through the alveolar border, one or both fangs might be removed at the same time; if not, they will be loosened in the socket, rendering their extraction comparatively easy with either the elevator or bent forceps.

*Dentes Sapientiæ.*—The first and second molars seldom present any difficulties in their removal from the irregularity of development; but the third molar, or dentes sapientiæ, are frequently only partially developed, and in such positions that present insurmountable obstacles to their extraction, either by our ordinary extracting forceps, or by the elevator. In many cases, in which there is not a sufficiency of room in the jaw to admit them, and from the continual suffering of the patient, the general health is likely to become impaired, and continue so from this source of local irritation—the extraction of the second molar becomes necessary after every other local remedy has failed, such as free incisions of the gum, leeches, poultices, &c.

In those cases where a *partial* development of the crown has taken place, and the remaining permanent teeth have been fully developed, and arranged in their proper position, leaving no room for the *dentes sapientiæ*—without crowding and disturbing the whole denture—the operation of extraction should be immediately performed, by first making a free incision of the gum *transversely*, at the posterior part of the tooth, and around its neck, and with the elevator firmly inserted between it and the second molar, the tooth can be gently raised from the socket, to allow of its removal by the ordinary forceps.

If not readily detached, the instrument should be forced backwards towards the posterior part of the jaw, for should any malformation of the fang exist, this action will not only materially assist in its removal, but will prevent undue laceration of the gum.

The annexed drawing represents a lower dens sapientiæ with a curved fang.

Again, these teeth will be found occasionally developed high up, and external to the alveolar ridge, the labial surface of the crown being only perceptible to the operator: in such cases the gum should be freely separated from the remaining part of the tooth, and the tooth either raised from the socket, or extracted with the elevator, in the same manner as in the case last described. When one of these teeth pierces the gum, on the inside of the alveolar ridge in the lower jaw—obstructing the tongue in its free action—and the crown has become sufficiently developed above the gum to admit of a firm grasp around its neck, the ordinary forceps are best adapted for its removal—using *less* internal lateral *than* external and perpendicular action.

Should, however, the crown be imperfectly developed through the gum, or destroyed by caries, I would recommend the pupil to freely scarify the gum around the tooth, and extract it with the elevator in the ordinary way: taking care, however, that the point of the instrument is inserted close to the second molar, and down to the edge of the alveolus, to prevent its slipping: to guard against which, the index finger of the other hand should be wrapped in a cloth, or protected with lint, and placed against the linguinal surface of the tooth, to act as a guard, and in case of slipping, to receive the point of the instrument, and thus prevent injury to the surrounding parts. There are also many other malformations of the fangs of teeth, which more or less impede their ready extraction; many of these are very difficult, and usually prolong the operation, and are generally perplexing and annoying to the student.

The following drawings exhibit a few of the cases in question:—

Fig. 6.—The roots of a first molar extracted from the lower jaw, after the crown had been broken off, and in which the fangs were curved and united at their apex, with an opening between, through which the transverse process passed.

Fig. 7.—Upper dens sapientiæ with three fangs—the palatine fang being curved.

Fig. 8.—Upper dens sapientiæ with four fangs.

Fig. 9.—Upper molar with four fangs, the buccal being united and curved at their apex.

Fig. 10.—Lower molar with diverging fangs.

Fig. 11.—Temporary lower molar with diverging fangs.

Fig. 12.—Upper dens sapientiæ with four fangs.

Fig. 6.

Fig. 7.

Fig. 8.

Fig. 9.

Fig. 10.

Fig. 11.

Fig. 12.

*N. B. The copyright of this and the three preceding articles are retained by the author.*

[*London Quart. Jour. Dent. Sci.*

## Figures and Tables

**Fig. 1. f1:**
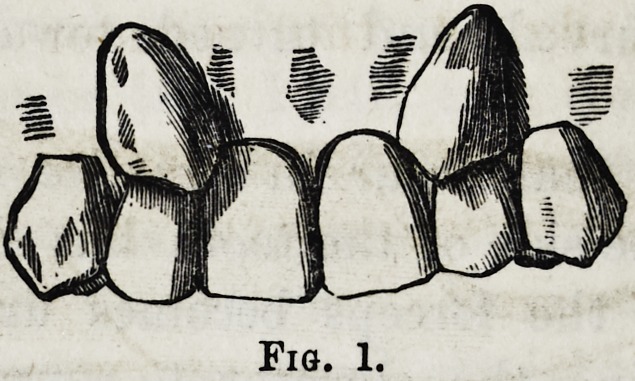


**Fig. 2. f2:**
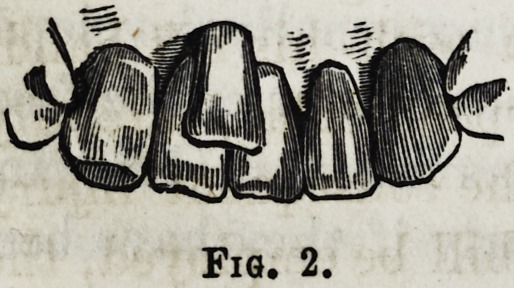


**Fig. 3. f3:**
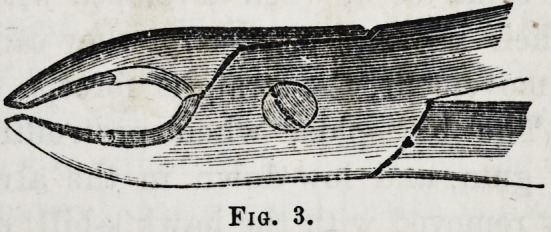


**Fig. 4. f4:**
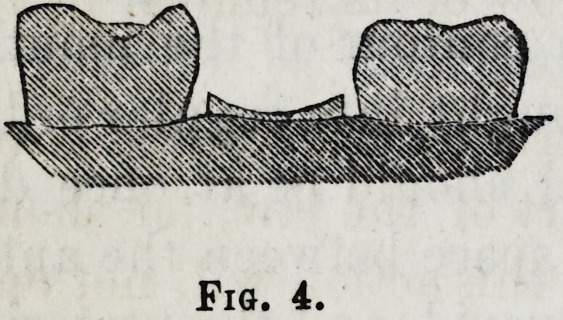


**Fig. 5. f5:**
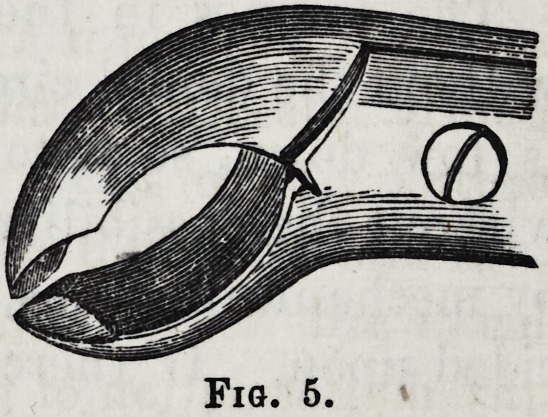


**Figure f6:**
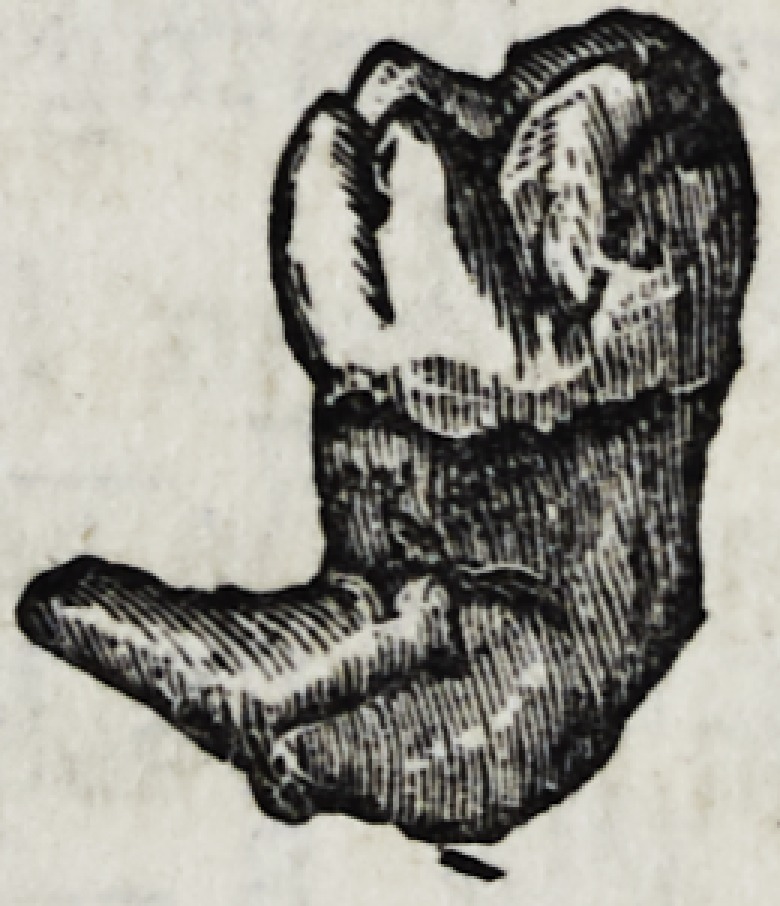


**Fig. 6. f7:**
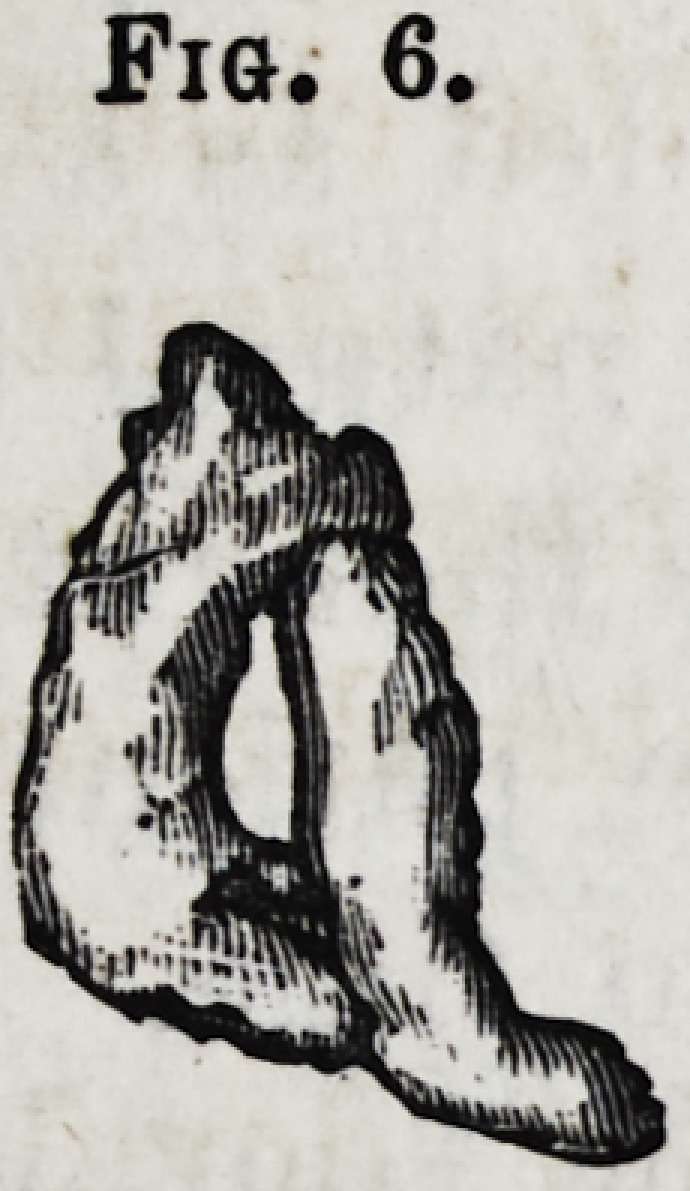


**Fig. 7. f8:**
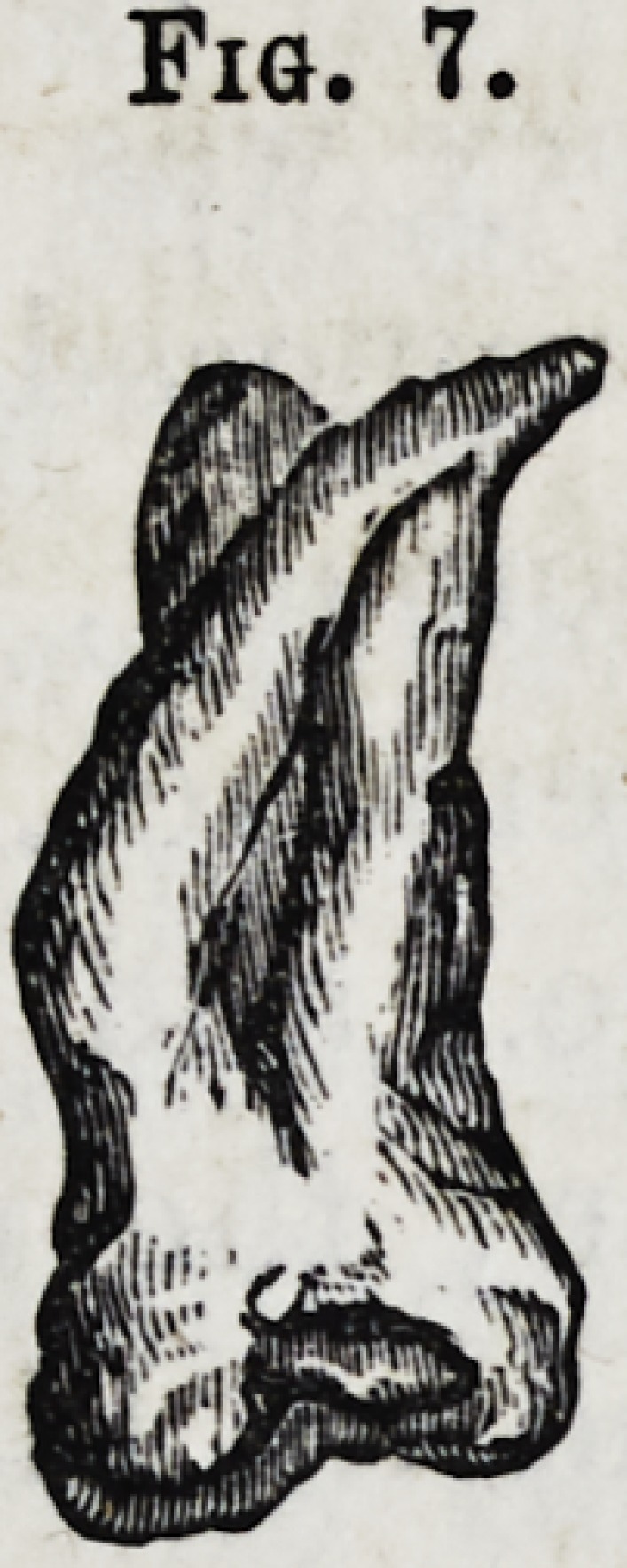


**Fig. 8. f9:**
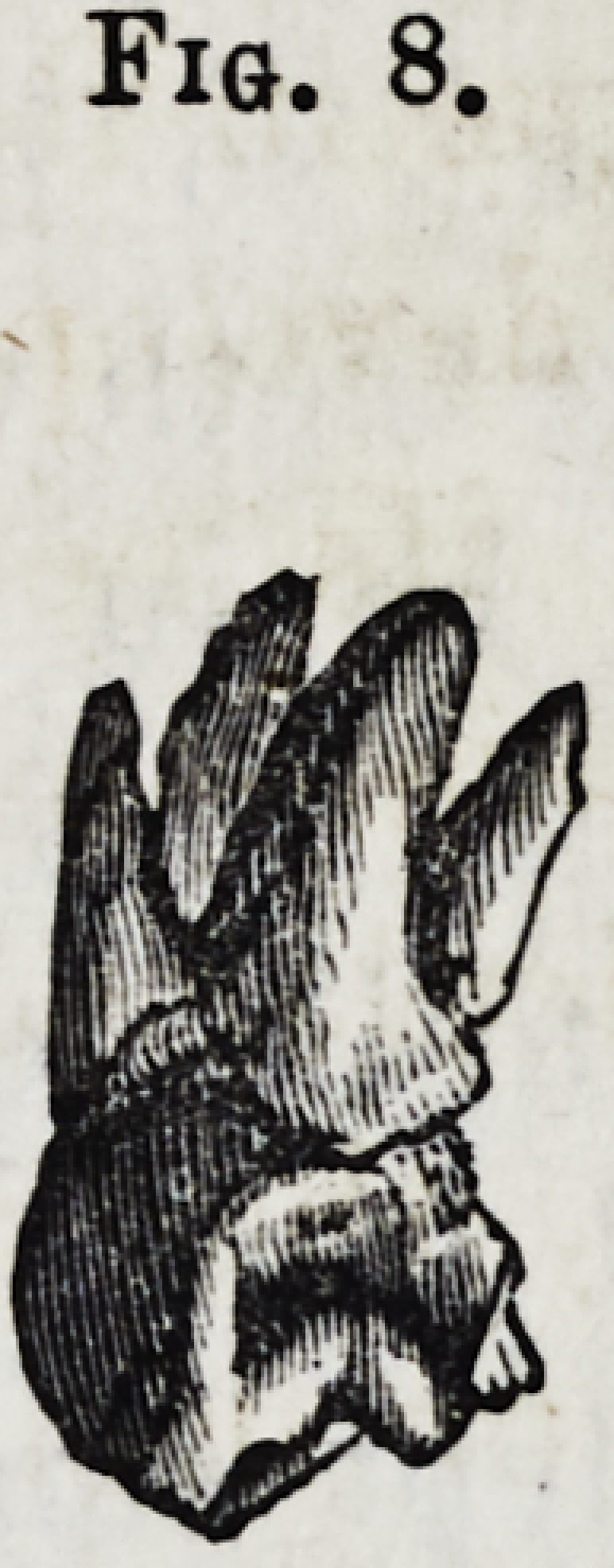


**Fig. 9. f10:**
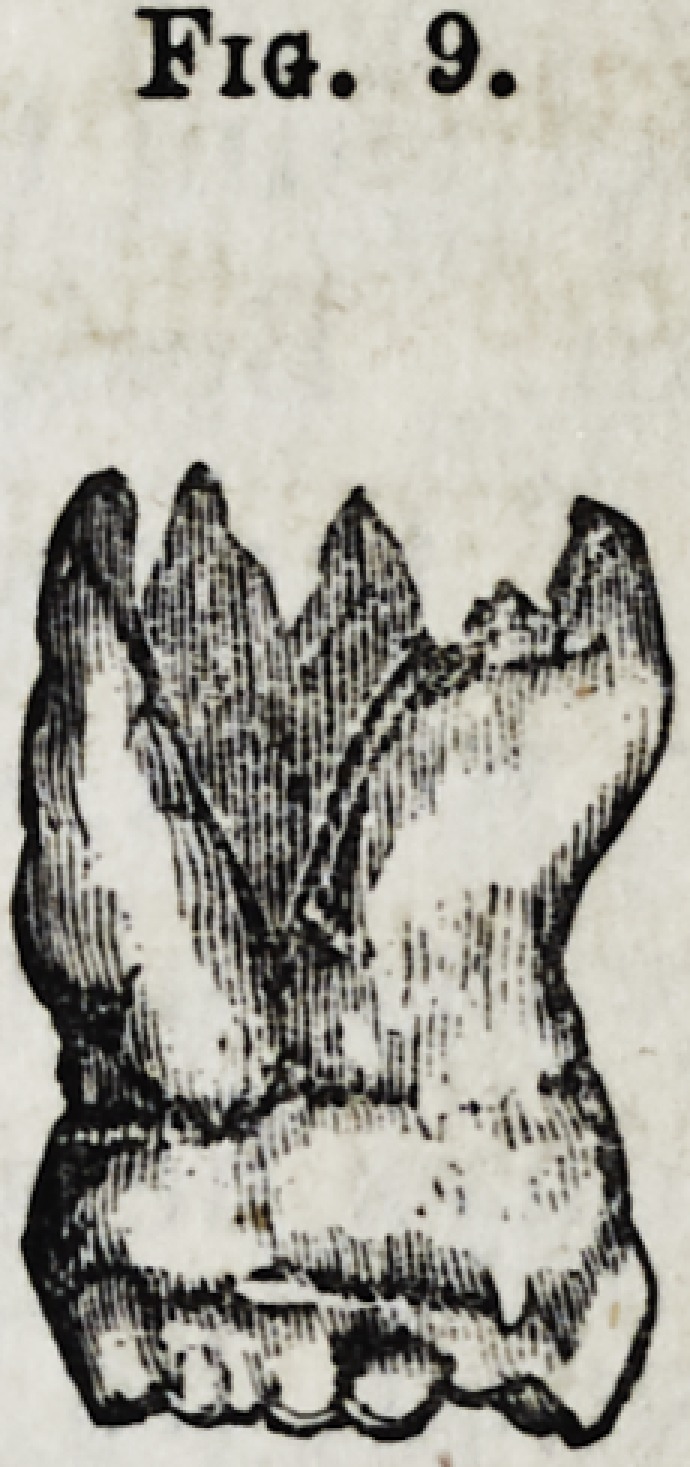


**Fig. 10. f11:**
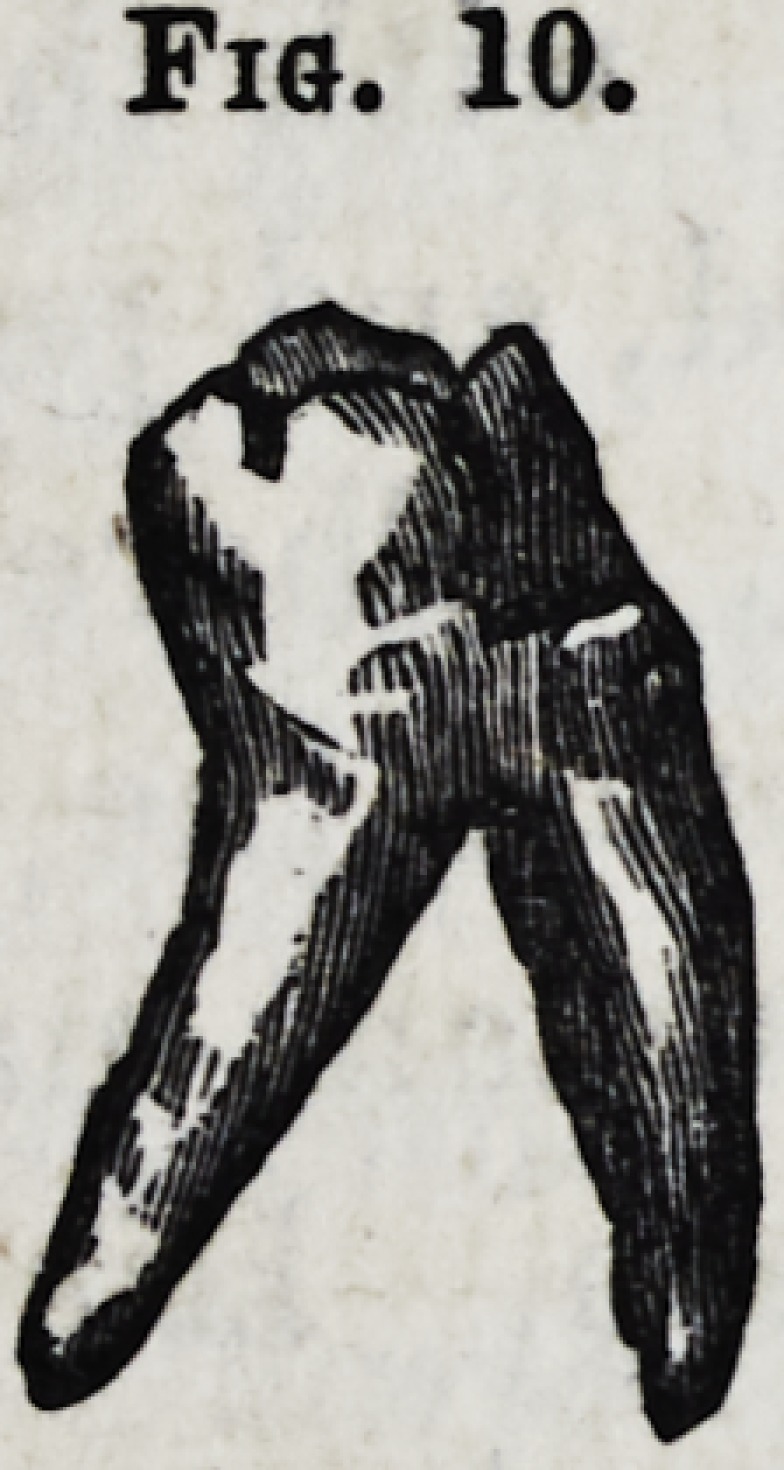


**Fig. 11. f12:**
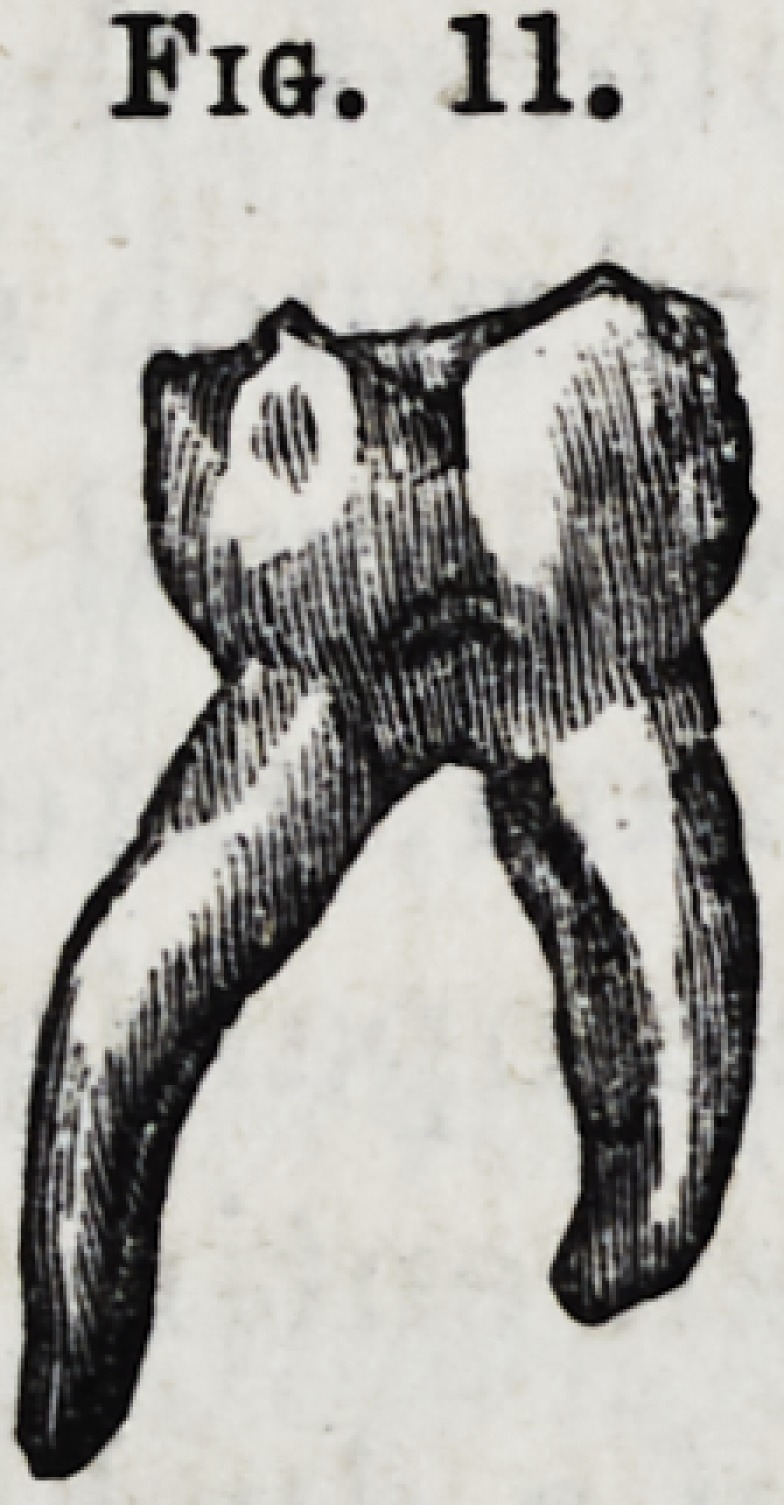


**Fig. 12. f13:**